# Adalimumab Is Associated With Lower Healthcare Resource and Steroid Use Versus Vedolizumab in Biologic-Naive Crohn’s Disease: A Retrospective Claims Database Analysis

**DOI:** 10.1093/crocol/otac029

**Published:** 2022-08-04

**Authors:** Ryan C Ungaro, Jenny Griffith, Viviana Garcia-Horton, Aolin Wang, Raymond K Cross

**Affiliations:** Division of Gastroenterology, Icahn School of Medicine at Mount Sinai, New York, New York, USA; AbbVie, Inc., North Chicago, Illinois, USA; Analysis Group, Inc., New York, New York, USA; Analysis Group, Inc., New York, New York, USA; Division of Gastroenterology & Hepatology, University of Maryland School of Medicine, Baltimore, Maryland, USA

**Keywords:** adalimumab, Crohn’s disease, healthcare resource use, vedolizumab

## Abstract

**Background:**

We compared real-world healthcare resource utilization (HRU), Crohn’s disease (CD)-related complications, and time to systemic corticosteroid discontinuation between patients with CD treated with adalimumab versus vedolizumab as initial biologic.

**Methods:**

Biologic-naïve adults with CD and ≥2 claims between 05/20/2014 and 09/30/2019 for adalimumab or vedolizumab were identified in the IBM MarketScan research database. Patient characteristics were assessed during the 6-month baseline period before biologic initiation (index date). Adalimumab- and vedolizumab-treated patients were propensity score-matched 1:1 on demographics, disease characteristics, and comorbidities with ≥10% prevalence that differed significantly between groups. Categorical, continuous, and time-to-event outcomes between groups during the 12-month follow-up on/after index were compared with chi-square tests, Wilcoxon rank-sum tests, and Kaplan–Meier analyses, respectively.

**Results:**

Adalimumab- and vedolizumab-treated patients were matched (*n* = 461 per group) and baseline characteristics balanced. Significantly fewer adalimumab- versus vedolizumab-treated patients had a CD-related emergency room visit (12-month proportion: 14.5% vs 21.0%; log-rank *P* < 0.01) or inpatient admission (14.9% vs 20.2%; log-rank *P* < 0.05). Rates of CD-related surgeries were similar (9.3% vs 11.5%; log-rank *P* = 0.282). Among patients without internal/perianal abscess or fistula or intestinal stricture at baseline (*N*_ADA_ = 360, *N*_VDZ_ = 364), numerically but not significantly fewer adalimumab- versus vedolizumab-treated patients had CD-related complications at 12 months (18.3% vs 22.3%; *P* = 0.171). Among patients with corticosteroid use at index (*N*_ADA_ = 143, *N*_VDZ_ = 139), significantly more adalimumab- versus vedolizumab-treated patients discontinued corticosteroids (12-month proportion: 90.2% vs 76.3%; log-rank *P* < 0.001).

**Conclusions:**

Patients with CD treated with adalimumab as their first biologic experienced significantly lower CD-related HRU and were more likely to discontinue corticosteroids compared to vedolizumab-treated patients.

## Introduction

Crohn’s disease (CD) is a chronic, idiopathic systemic inflammatory disease primarily affecting the gastrointestinal tract and is characterized by skip lesions and transmural inflammation.^1^ Hospitalization and surgery are major events in the natural history of CD, and are important predictors of morbidity in the disease.^2^ Previous studies have reported that between 52% and 66% of patients with CD in the United States (US) and Canada were hospitalized over 10–15 years follow-up, and the rate of CD-related hospitalizations in the US has significantly increased over time.^3,4^ Early CD-related hospitalization is predictive of the need for surgery,^5^ which will eventually be required for as many as 60%–80% of patients with CD.^2,6,7^

However, disease recurrence following surgery is common,^7^ leading to high healthcare resource utilization (HRU) and costs over a patient’s lifetime.^8–10^ The mean direct all-cause per-patient-per-year healthcare costs among patients with CD have been rising since 2013 compared to matched controls.^8,10^ Inpatient and surgical hospitalizations heavily contribute to the overall costs of CD in the United States, accounting for 53%–67% of direct CD-related medical costs,^11,12^ with higher costs among patients with severe and worsening CD.

The clinical management of CD depends on disease symptoms and severity, and corticosteroids may help treat moderate-to-severe symptom flares.^13^ However, even short-term use may lead to systemic adverse events (ie, bone loss, mood disorder, insomnia, hypertension, elevated blood glucose, narrow angle glaucoma, acne, weight gain, and hypoadrenalism), potentially irreversible toxicities, steroid dependency, or complications such as abscess and fistula.^13–16^ Thus, the American College of Gastroenterology (ACG) treatment guidelines recommend that corticosteroids be used sparingly and discontinued as soon as possible.^13^

Adalimumab, a fully human anti-tumor necrosis factor (TNF) monoclonal antibody, and vedolizumab, a humanized anti-α4β7 integrin monoclonal antibody, are both US Food and Drug Administration (FDA)-approved biologic treatments for moderate-to-severe CD.^17,18^ In separate placebo-controlled trials, adalimumab (CHARM^19^) and vedolizumab (GEMINI 2^20^) have been shown to improve clinical outcomes and maintain remission in biologic-naive patients with moderate-to-severe CD. Network meta-analyses have been conducted to assess comparative efficacy and safety of biologics in patients with moderate-to-severe CD,^21^ and adalimumab has been compared in head-to-head randomized trials against ustekinumab in CD^22^ and vedolizumab in ulcerative colitis (UC).^23^ However, there are no head-to-head randomized controlled trials directly comparing adalimumab and vedolizumab treatment in CD.

Real-world data on the comparative effectiveness of adalimumab versus vedolizumab as first-line biologic on the clinical and economic burdens of CD are sparse, although such data would be valuable for treatment decision-making. To address this knowledge gap, we analyzed a large health claims database to compare all-cause and CD-related HRU and costs (primary outcomes), and CD-related complications (any internal fistula/abscesses, perianal fistulas/abscesses, intestinal strictures, hospitalization, or surgery for CD) and time to systemic corticosteroid discontinuation (secondary outcomes), between patients with CD treated with adalimumab versus vedolizumab as initial biologic.

## Materials and Methods

### Data Source

This retrospective claims analysis used data from the IBM MarketScan Research Database (01/01/2000–09/30/2019).^24^ This database contains private sector healthcare data of employees and dependents covered by the health benefit programs of 160+ contributing US employers. Enrollment history and claims for medical (provider and institutional) and pharmacy services are collected from 40+ health plans and represent 263+ million unique patients since 1995. Inpatient services are recorded at the individual claim and summarized stay levels.

As this analysis used de-identified claims data, no institutional board review was required.

### Sample Selection

#### Inclusion criteria

To be included in the study, patients were required to have ≥2 claims with a diagnosis of CD on separate days (International Classification of Diseases, Ninth/Tenth edition, Clinical Modification codes: 555.xx and K50.xx). Patients were additionally required to be at least 18 years old at the *index date* (defined as the date of the first claim for adalimumab or vedolizumab on or after May 20, 2014, the date of FDA approval of vedolizumab in CD); have continuous eligibility in their medical and pharmacy benefits for ≥6 months before (*baseline period*) and ≥12 months after the index date (*follow-up period*); have ≥2 claims for the index treatment on separate days; and have ≥1 claim with a CD diagnosis in the baseline period.

#### Exclusion criteria

Patients were excluded if they used any other non-index biologic treatments approved by the FDA for CD (ie, certolizumab pegol, infliximab, natalizumab, or ustekinumab) before the index date, or if they had autoimmune diseases (other than CD) for which adalimumab is indicated during the baseline period (ie, rheumatoid arthritis, psoriasis, psoriatic arthritis, ankylosing spondylitis, non-infectious uveitis, or hidradenitis suppurativa). Patients who had ≥2 claims with a diagnosis of UC and those with an equal or higher number of UC versus CD claims were excluded to increase the likelihood that selected patients had CD.^25–29^ Patients with managed care plans (not fee for service) were excluded as their cost data may be incomplete.

#### Patient cohorts

Two patient cohorts were defined according to the first observed biologic treatment for CD (identified as described in Supplementary Table S1) (index treatment): adalimumab-treated patients and vedolizumab-treated patients.^30^ Biologic transitions were allowed after index.

#### Matching

Greedy matching via propensity scores (with a caliper of 0.25 of the standard deviation [SD] of the propensity score) was used to match adalimumab- to vedolizumab-treated patients 1:1. The propensity score was estimated based on demographics (ie, age at index, sex, region, index year, insurance type); comorbidity profile (Charlson Comorbidity Index [CCI], abdominal and pelvic symptoms, noninfectious gastroenteritis and colitis, hypertension, respiratory or other chest symptoms, anxiety, hyperlipidemia, malignant neoplasms, abdominal pain, diarrhea, rectal bleeding, fistula, stricture); treatment history (any use of systemic corticosteroids, opioids, immunosuppressants, prescription nonsteroidal anti-inflammatory drugs [NSAIDs], and aminosalicylates); any disease-related inpatient admission, gastroenterologist visit, or disease-related surgery during baseline; number of CD-related outpatient and emergency room (ER) visits during baseline; and all-cause treatment costs during baseline.

### Study Variables

#### Baseline demographic and clinical characteristics

Demographic characteristics were measured at the index date. Comorbidity profile (CCI and select additional comorbidities [Table 1]); disease characteristics and CD location at index; CD treatments (systemic corticosteroids, opioids, aminosalicylates, or immunomodulators); all-cause and CD-related HRU; and all-cause treatment costs were measured during the six-month baseline period.

**Table 1.** Patient characteristics after matching.^a^

	Adalimumab *N* = 461	Vedolizumab *N* = 461
**Demographics as of the index date**		
Age, mean ± SD (years)	43.30 ± 15.01	43.34 ± 14.09
Male, *n* (%)	216 (46.9%)	210 (45.6%)
US region of residence, *n* (%)		
Northeast	75 (16.3%)	99 (21.5%)
North Central	115 (24.9%)	116 (25.2%)
South	196 (42.5%)	185 (40.1%)
West	75 (16.3%)	60 (13.0%)
CCI, mean ± SD	0.50 ± 1.06	0.52 ± 1.09
**Disease characteristics during the baseline period, *n* (%)**		
Abdominal pain	200 (43.4%)	209 (45.3%)
Anemia	104 (22.6%)	115 (24.9%)
Anxiety	71 (15.4%)	80 (17.4%)
Diarrhea	142 (30.8%)	150 (32.5%)
Rectal bleeding	55 (11.9%)	56 (12.1%)
Respiratory or other chest symptoms	106 (23.0%)	100 (21.7%)
Gastrointestinal hemorrhage	35 (7.6%)	31 (6.7%)
Symptoms of the abdomen and pelvis	241 (52.3%)	247 (53.6%)
CD-related complications (any)	101 (21.9%)	97 (21.0%)
Stricture (intestinal)	63 (13.7%)	63 (13.7%)
Perianal fistula	22 (4.8%)	25 (5.4%)
Perianal abscess	20 (4.3%)	13 (2.8%)
Internal fistula	15 (3.3%)	15 (3.3%)
Internal abscess	3 (0.7%)	3 (0.7%)
CD location as of the index date		
Small intestine	131 (28.4%)	93 (20.2%)
Large intestine	80 (17.4%)	89 (19.3%)
Small and large intestine	87 (18.9%)	88 (19.1%)
Unspecified	177 (38.4%)	199 (43.2%)
**Treatment history during the baseline period, *n* (%)**		
Systemic corticosteroids	243 (52.7%)	263 (57.0%)
Opioids	181 (39.3%)	184 (39.9%)
Aminosalicylates	105 (22.8%)	113 (24.5%)
Immunosuppressants	92 (20.0%)	102 (22.1%)
**HRU during the baseline period**		
All-cause HRU: proportion of patients with any, *n* (%)		
Inpatient admissions	99 (21.5%)	109 (23.6%)
ER visits	130 (28.2%)	148 (32.1%)
CD-related HRU: proportion of patients with any, *n* (%)		
Inpatient admissions	90 (19.5%)	100 (21.7%)
ER visits	49 (10.6%)	66 (14.3%)
**All-cause treatment costs (2020 US dollars) during the baseline period**		
Mean ± SD	$2801 ± $6187	$3661 ± $7139
Median	926	1314
IQR	(132, 3213)	(105, 4097)

Abbreviations: CCI, Charlson Comorbidity Index; CD, Crohn’s disease; ER, emergency room; HRU, health resource utilization

All baseline characteristics were balanced between cohorts (standardized mean differences <0.2).

#### Outcomes assessed during the follow-up period

All-cause and CD-related HRU were assessed from the index date (inclusive) to first occurrence of each ER visit, inpatient admission, and CD-related surgery. Sensitivity analyses that assessed cumulative all-cause and CD-related HRU over the entire follow-up period were also performed. CD-related HRU was identified from medical service claims with a primary or secondary diagnosis code for CD.

The time from the index date to first CD-related complication (ie, internal fistulas or abscesses, perianal fistulas or abscesses, intestinal strictures, hospitalization for CD, or surgery for CD [see Supplementary Table S2 for codes]), and the proportions with each complication, were reported among patients without any fistula, abscess, or intestinal stricture during the baseline period.

Among patients with concomitant systemic corticosteroid use at the index date, time to discontinuation (the time from the index date to the end date of a systemic corticosteroid episode with no subsequent prescription fills within 60 days) was reported.

All-cause and CD-related healthcare payments to the provider were calculated and included medical service costs (ie, the sum of hospitalization, ER, and outpatient costs, excluding claims associated with patients’ index treatment) and treatment costs (ie, the sum of index treatment costs and other pharmacy costs). Index treatment costs included pharmacy and medical service claims associated with index treatments. All-cause costs included medical costs incurred for any cause regardless of diagnosis and all pharmacy claims. CD-related medical costs were those for medical services with a diagnosis code for CD, and CD-related treatment costs were those with the index treatment or any CD-related treatments (ie, immunosuppressants, systemic corticosteroids, NSAIDs, aminosalicylates, or opioids). Costs were adjusted to 2020 US dollars using the medical care component of the Consumer Price Index.^31^

### Statistical Analysis

Descriptive statistics were used to summarize baseline patient characteristics and outcomes in the follow-up period. Statistical comparisons between propensity score-matched cohorts were conducted using chi-square tests (or Fisher’s exact tests for expected counts <10), Wilcoxon rank-sum tests, and Kaplan–Meier (KM) analysis with log rank tests for categorical, continuous, and time-to-event outcomes, respectively. All analyses were conducted using SAS Enterprise Guide version 7.1 (SAS Institute Inc., Cary, North Carolina, US). Statistical significance was based on a two-sided alpha level of 0.05.

## Results

### Baseline Characteristics

After matching, 461 adalimumab-treated patients and 461 vedolizumab-treated patients were included in the sample (Supplementary Figure S1). All baseline characteristics were balanced between cohorts (standardized mean differences <0.2^32^) (Table 1; Supplementary Table S3). In both cohorts, the mean age was 43 years, 46%–47% were male, and 40%–43% were from the South. The mean CCI was 0.5 in both cohorts; common CD comorbidities were anemia (23%–25%), respiratory or other chest symptoms (22%–23%), and anxiety (15%–17%). During the baseline period, 21%–22% experienced CD-related complications (ie, internal abscess [0.7%] or fistula [3%], perianal abscess [3%–4%] or fistula [5%], or intestinal stricture [14%]). Over half of patients (53%–57%) used systemic corticosteroids and 40% of both cohorts used opioids.

During the baseline period, 22%–24% of patients experienced an all-cause inpatient admission, the majority of which were CD related, and 28%–32% had all-cause ER visits, with approximately one-third to half being CD related. The average all-cause treatment costs during the baseline period were $2801 (SD: $6187) for adalimumab-treated patients and $3661 ($7139) for vedolizumab-treated patients.

### HRU During Follow-up

**Figure 1.**
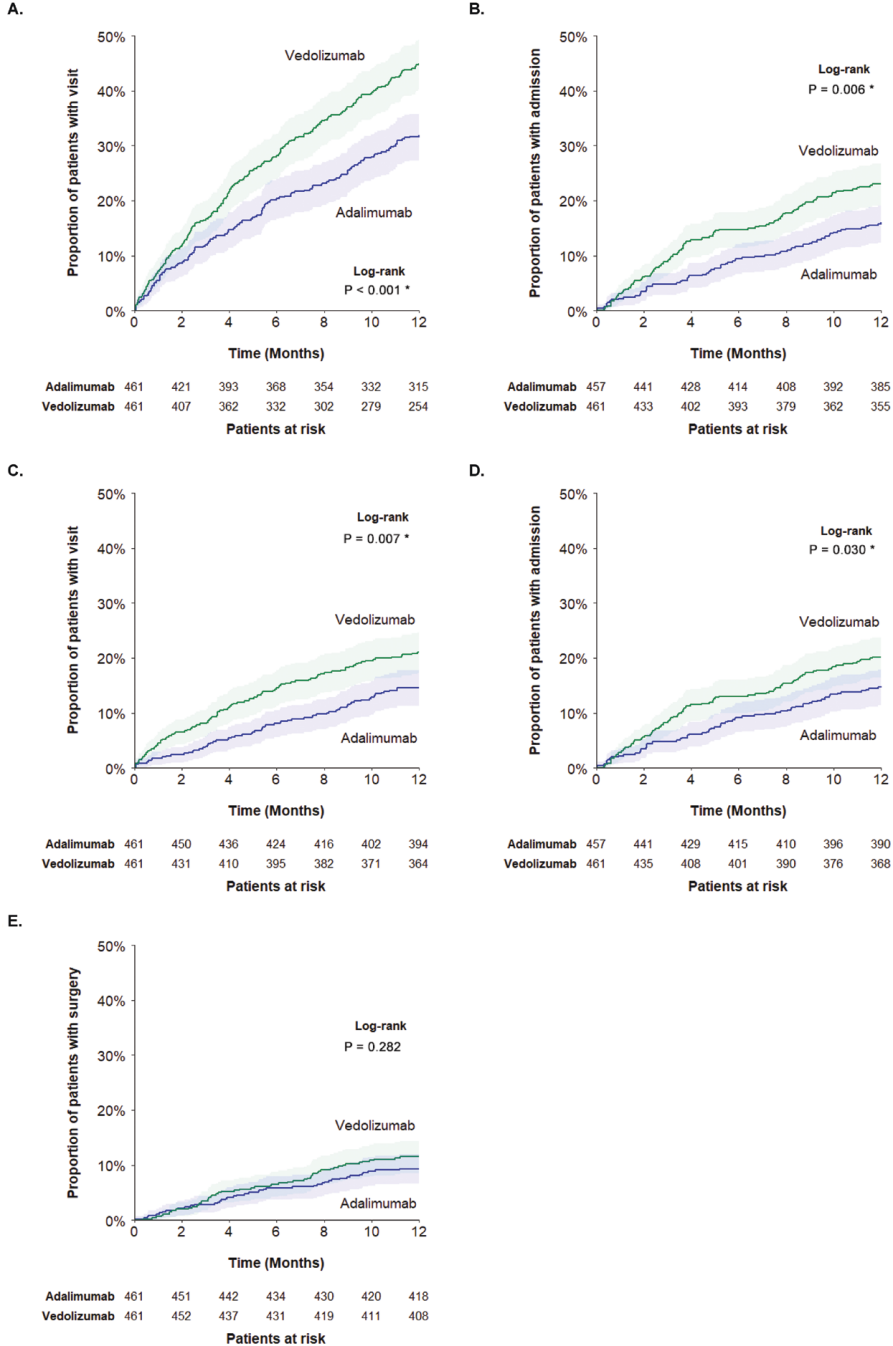
Health resource utilization during the follow-up period. A, Time to first all-cause emergency room visit. B, Time to first all-cause inpatient admission. C, Time to first CD-related emergency room visit. D, Time to first CD-related inpatient admission; E. Time to first CD-related surgery. Abbreviation: CD, Crohn’s disease.

During the 12-month follow-up period, significantly fewer adalimumab-treated patients experienced an all-cause ER visit (proportion at 12 months: 31.9% vs 44.9%; log-rank *P* < 0.001; Figure 1A) or inpatient admission (16.0% vs 23.0%; log-rank *P* = 0.006; Figure 1B) compared to vedolizumab-treated patients. Likewise, significantly fewer adalimumab-treated patients experienced a CD-related ER visit (proportion at 12 months: 14.5% vs 21.0%; log-rank *P* = 0.007; Figure 1C) or inpatient admission (14.9% vs 20.2%; log-rank *P* = 0.030; Figure 1D) compared to vedolizumab-treated patients. In addition, vedolizumab-treated patients experienced their first ER visit and inpatient admission (both all-cause and CD-related) earlier in the follow-up period compared with adalimumab-treated patients. There were no significant differences between adalimumab- and vedolizumab-treated patients in the proportion with a CD-related surgery (proportions at 12 months: 9.3% vs 11.5%; log-rank *P* = 0.282) (Figure 1E). Analyses that assessed cumulative all-cause and CD-related HRU over the 12-month follow-up period revealed similar results: on average, adalimumab-treated patients experienced significantly lower numbers of all-cause and CD-related inpatient admissions and ER visits compared to vedolizumab-treated patients (all *P* < 0.05) (Supplementary Table S4).

### CD-Related Complications During the Follow-up Period

**Figure 2.**
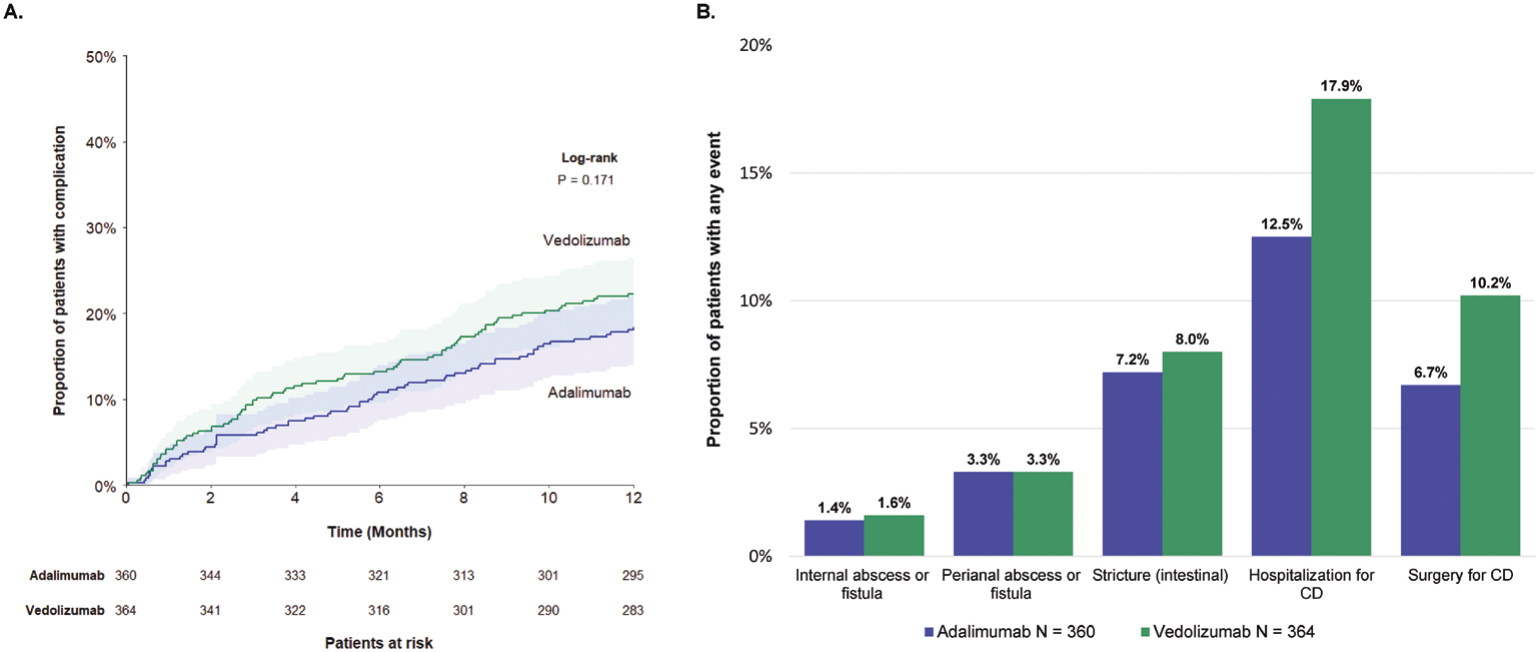
CD-related complications during the follow-up period. A, Time to first CD-related complication. B, Proportion of patients with any CD-related complications. Abbreviation: CD, Crohn’s disease.

CD-related complications were evaluated among the 360 adalimumab-treated patients and 364 vedolizumab-treated patients without any diagnoses of internal abscess or fistula, perianal abscess or fistula, or intestinal stricture during the 6-month baseline period. During the follow-up period, there were no significant differences between patients treated with adalimumab or vedolizumab in the time to first CD-related complication (proportions at 12 months: 18.3% vs 22.3%; log-rank *P* = 0.171; Figure 2A) or across individual CD-related complications (ie, 1.4% vs 1.6% for internal abscess or fistula; both 3.3% for perianal abscess or fistula; 7.2% vs 8.0% for intestinal stricture; 6.7% vs 10.2% for CD-related surgery; 12.5% vs 17.9% for CD-related hospitalization; all *P* > 0.05) (Figure 2B).

### Time to Corticosteroid Discontinuation During the Follow-up Period

**Figure 3.**
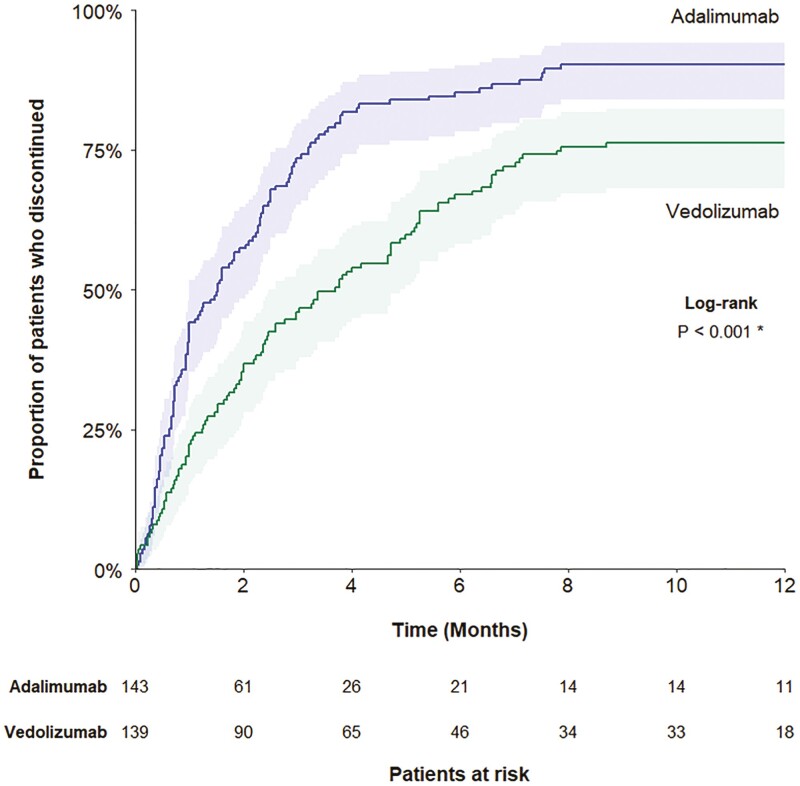
Time to corticosteroid discontinuation in the follow-up period. * denotes statistical significance. Abbreviation: CD, Crohn’s disease.

Time to systemic corticosteroid discontinuation was evaluated among the 143 adalimumab-treated patients and 139 vedolizumab-treated patients using systemic corticosteroids at index treatment initiation. A significantly higher proportion of adalimumab-treated patients discontinued corticosteroids during follow-up compared with vedolizumab-treated patients (proportions at 12 months: 90.2% vs 76.3%; log-rank *P* < 0.001) (Figure 3). Furthermore, the median time to discontinuation was significantly shorter for patients treated with adalimumab vs vedolizumab who did discontinue corticosteroid use (1.53 vs 3.70 months; log-rank *P* < 0.001).

### Healthcare Costs During the Follow-up Period

**Figure 4.**
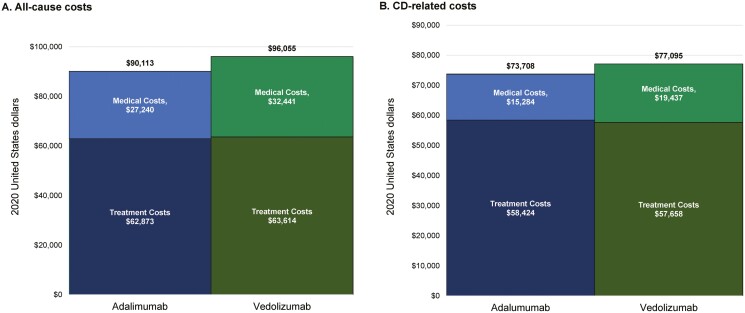
Healthcare costs in the follow-up period. A, All-cause costs; B. CD-related costs. Abbreviation: CD, Crohn’s disease.

Figure 4 illustrates the mean all-cause (Figure 4A) and CD-related (Figure 4B) healthcare costs among the cohorts, overall and by component (medical and treatment). During the follow-up period, adalimumab-treated patients had significantly lower mean all-cause medical costs ($27 240 vs $32 441; *P* < 0.001), all-cause treatment costs ($62 873 vs $63 514; *P* = 0.008), and CD-related medical costs ($15 284 vs $19 437; *P* < .001) compared with those treated with vedolizumab. Adalimumab-treated patients had significantly higher mean CD-related treatment costs ($58 424 vs $57 658; *P* = 0.002) than vedolizumab-treated patients. The mean total all-cause healthcare costs ($90 113 vs $95 955; *P* = 0.466) and mean total CD-related healthcare costs ($73 707 vs $77 095; *P* = 0.349) were not significantly different between cohorts. The full distribution of all-cause and CD-related healthcare costs can be found in Supplementary Table S4.

## Discussion

This real-world study compared HRU, emergent CD-related complications, and time to corticosteroid discontinuation among patients with moderate-to-severe CD treated with adalimumab vs vedolizumab as their first biologic. Over the 12-month follow-up period, the proportions of patients with all-cause and CD-related ER visits and inpatient admissions were significantly lower among patients treated with adalimumab compared to vedolizumab. In addition, among those taking corticosteroids at the index date, adalimumab-treated patients were significantly more likely to discontinue corticosteroids and discontinue earlier during follow-up than those on vedolizumab. While adalimumab-treated patients had numerically lower proportions with CD-related surgeries and complications compared with vedolizumab-treated patients (among those without CD-related complications during baseline), there were no significant differences.

There are no head-to-head randomized controlled trials comparing the efficacy and safety of adalimumab vs vedolizumab in CD and limited real-world data exists. A real-world study by Macaluso et al on clinical remission rates at 12 and 52 weeks among patients with CD reported that both adalimumab and vedolizumab had comparable effectiveness and safety profiles. However, the patient population in Macaluso et al was from a regional group of centers specializing in patients with inflammatory bowel disease and may not be representative of the general patient population. A real-world study by Bohm et al reported that steroid-free clinical remission rates were not significantly different between anti-TNF-naÏve patients with CD treated with vedolizumab or anti-TNF therapies.^33^ That study did not examine HRU among patients with CD. Singh et al conducted a systematic review and network meta-analysis of phase 2 and 3 randomized controlled trials comparing first- and second-line biologic therapies for moderate-to-severe CD and reported that treatment with adalimumab or infliximab consistently ranked highly for induction and maintenance of clinical remission.^21^ A systematic review and meta-analysis by Mao et al noted statistically significant reductions in the likelihood of hospitalizations and surgery among patients with CD or UC treated with adalimumab but not with vedolizumab in randomized controlled trials. However, the data were limited in that study (eg, only 1 vedolizumab trial was included) and no real-world comparisons were conducted. Nevertheless, Mao et al’s findings on adalimumab are supported by a post hoc analysis of CHARM clinical trial data which reported that the one-year risk of hospitalization and major CD-related surgery were significantly lower among adalimumab-treated patients compared to those receiving placebo.^34^

Our finding that adalimumab-treated patients incurred lower CD-related HRU compared to vedolizumab-treated patients is consistent with the findings reported in existing literature, and may be partially attributable to the superiority of anti-TNFs versus α_4_β_7_ integrin blockers in healing small bowel for patients with CD.^35^ Despite both adalimumab and vedolizumab effectively leading to endoscopic remissions in moderate-to-severe inflammatory bowel disease (IBD), different findings have been reported in CD versus UC. The VARSITY trial^23^ showed superiority of vedolizumab over adalimumab in UC regarding endoscopic improvement at week 52. On the other hand, a 2022 post-hoc analysis of pooled data from patients with moderate-to-severe CD from four clinical trials reported superior rates of one-year endoscopic healing in adalimumab-treated and infliximab-treated patients than vedolizumab-treated patients.^35^

Previous studies have presented some evidence suggesting that adalimumab may outperform vedolizumab at achieving corticosteroid-free remission for patients with CD, but differences were not statistically significant. The real-world, propensity score weighted study by Macaluso et al reported that the proportion of patients with steroid-free remission was numerically, but not significantly, higher among the adalimumab-treated vs vedolizumab-treated cohorts.^36^ A retrospective observational cohort study using data from a North America-based consortium registry found that steroid-free clinical remission was not statistically different between anti-TNF-naÏve patients treated with vedolizumab or anti-TNF therapy (infliximab, adalimumab, or certolizumab).^33^ Conversely, the current study found that adalimumab-treated patients were significantly more likely to discontinue corticosteroids during the 12-month follow-up period, and to spend less time on corticosteroids. This finding has important clinical implications given that the ACG treatment guidelines for CD recommend that corticosteroids should be discontinued as soon as possible due to risk of steroid dependency, abscess, fistula, and systemic adverse events.^13^

The present cohort of patients with CD appears to have similar characteristics to what would be expected in the general population with CD. Approximately 21%–22% of the current sample had any CD-related complications at baseline. The rates of intestinal stricture (14%), perianal fistula (5%), perianal abscess (3%–4%), internal fistula (3%), or internal abscess (0.7%) are expected given the current cohort is biologic-naÏve and likely at an earlier point in their disease course, but are lower than previously published rates. A multi-country, retrospective chart review study reported that prevalence of active fistula at index was 16.7% and 4.2% for biologic-naÏve patients with CD treated with anti-TNF treatments and vedolizumab, respectively. In population-based cohorts, the frequency of perianal fistulas was 10%–26%,^37,38^ and the cumulative risk has been estimated at 26% at 20 years after diagnosis.^13^ The current study observed no significant differences between adalimumab and vedolizumab in the incidence of these CD complications during follow-up. However, the relative and cumulative impact of biologics on these complications may have been hard to detect among patients with low risk and a limited 12-month follow-up.

This study benefits from several important strengths. The use of the MarketScan claims databases permitted the selection of a large sample of patients with CD for the real-world analyses of the impact of first-line adalimumab vs vedolizumab on HRU and systemic corticosteroid use. Additionally, the propensity score matching design that accounted for the differences in key baseline characteristics between cohorts allowed fairer comparisons between adalimumab- and vedolizumab-treated patients. The results of this study should also be considered in light of several limitations, some of which are common to retrospective claims database analyses (possible coding errors or omissions of claims). For example, CD location is subject to misclassification and a large proportion of this data was missing. Certain comorbidities (eg, fistula/stricture) may have been used by physicians to decide whether to prescribe adalimumab or vedolizumab but may be underestimated due to coding incompleteness/inaccuracies/misclassification. These factors may not have been accounted for through the propensity score matching, but there was no reason to believe that differences in coding practices existed between the two cohorts. Information used to identify patients’ first use of adalimumab or vedolizumab was based on a baseline period of ≥6 months due to sample size considerations; misclassification of prevalent cases as incident cases may be possible. Despite the use of a modal approach to reclassify patients with both CD and UC diagnoses, it is still possible that some patients with UC were included in the sample. This may bias the results in favor of vedolizumab, as higher clinical remission rates were found among patients treated with vedolizumab than with adalimumab in the VARSITY trial.^23^ Additionally, there may be remaining confounding after matching due to characteristics like disease severity that may not be as accurately defined in this type of dataset. Clinical outcomes cannot be directly validated in deidentified claims due to the lack of electronic health records. Similarly, a measure of medication supply is not available in medical claims, and hence the imputed corticosteroid use of 1 day for medical claims in the current study may be subject to measurement error. The reimbursed pharmacy costs captured in the data do not reflect the actual drug costs, as rebating is not included and therefore may be overestimated. The time to corticosteroid discontinuation analysis may be subject to censorship bias due to limited follow-up. HRU and healthcare costs were assessed for each cohort over the entire follow-up period, regardless of whether or not patients discontinued the index treatment. Patients treated with vedolizumab were included from the time of its approval, and there may be a learning curve for its use by physicians (eg, timing of steroid tapering, dose escalation). Finally, this cohort was drawn from a convenience sample of a commercially insured population; thus, the results of this study may not be generalizable to all patients with moderate-to-severe CD, such as patients with Medicare, Medicaid, or the uninsured.

## Conclusions

This real-world study found that patients with CD who received adalimumab as their first biologic treatment were significantly less likely to experience CD-related HRU and more likely to discontinue corticosteroids compared to those who took vedolizumab, with no significant differences in average all-cause or CD-related costs. Our results suggest that, compared to vedolizumab, adalimumab may be associated with better overall outcomes among anti-TNF-naÏve patients with CD. These results should be interpreted within the context of observational studies where residual confounding may exist and confirmed in future research using other prospective datasets.

## Supplementary Material

otac029_suppl_Supplementary_Material

## Data Availability

Data were not publicly available.

## Writing Assistance

Medical writing assistance was provided by Shelley Batts, PhD, an employee of Analysis Group, Inc. Support for this assistance was provided by AbbVie, Inc.
