# Supplementary material for: Adalimumab Is Associated With Lower Healthcare Resource and Steroid Use Versus Vedolizumab in Biologic-Naive Crohn’s Disease: A Retrospective Claims Database Analysis
Source: Crohns Colitis 360. 2022 Aug 4;4(3):otac029. doi: 10.1093/crocol/otac029 (PMC9434638; doi:10.1093/crocol/otac029)
Supplement: otac029_suppl_Supplementary_Material [file otac029_suppl_supplementary_material.docx]

**Adalimumab is associated with lower healthcare resource and steroid use versus vedolizumab in biologic-naive Crohn’s disease: A retrospective claims database analysis**

**SUPPLEMENTAL MATERIALS**

**Supplemental Figure 1. Sample selection^a^**

**
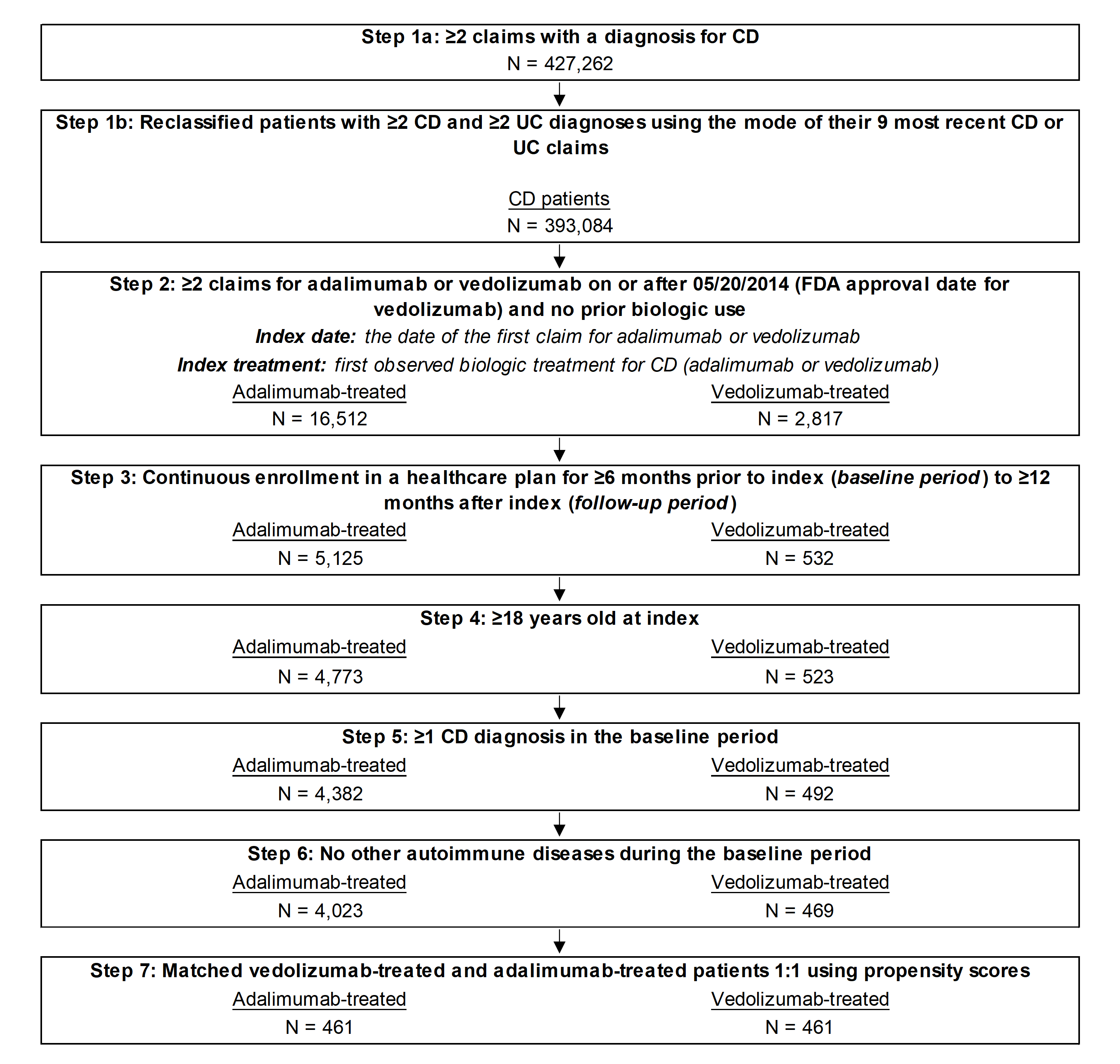
**

**Abbreviations:** CD, Crohn's disease; FDA: Food and Drug Administration; ICD-9/10-CM: International Classification of Diseases, Ninth/Tenth edition, Clinical Modification; UC: ulcerative colitis.

**Note**: ^a^ The codes used to identify CD were 555.xx (ICD-9-CM) and K50.xx (ICD-10-CM). The codes used to identify UC were 556.xx (ICD-9-CM) and K51.xx (ICD-10-CM).

**Supplemental Table 1. Adalimumab and vedolizumab codes**

| Drug | General Product Identifier (GPI) | Healthcare Common Procedure Coding System (HCPCS) | National Drug Code (NDC) |
| --- | --- | --- | --- |
| Adalimumab | 6627001500 | J0135 |  |
| Vedolizumab^a^ |  | C9026 or J3380 | 64764030020 |

**Abbreviations:** CD, Crohn's disease; UC: ulcerative colitis; USD, US dollars.

**Note**: ^a^ Because the vedolizumab-specific HCPCS codes were issued on January 1, 2016, pre-2016 claims were classified as vedolizumab treatment if they had an unclassified drug or biologic code (HCPCS: J3590, J3490, or C9399), along with a CD or UC diagnosis code and a payment amount of at least $4,500 (2015 USD) per Dubinsky et al. (Dubinsky MC, Cross RK, Sandborn WJ, et al. Extraintestinal manifestations in vedolizumab and anti-TNF-treated patients with inflammatory bowel disease. Inflamm Bowel Dis. 2018;24(9):1876-82.)

**Supplemental Table 2. Gastrointestinal surgery codes**

| Code | Description | | | |
| --- | --- | --- | --- | --- |
| **Current Procedural Terminology (CPT)** | | | | |
| 44120 | Enterectomy, resection of small intestine; single resection and anastomosis | | | |
| 44121 | Enterectomy, resection of small intestine; each additional resection and anastomosis (List separately in addition to code for primary procedure) | | | |
| 44125 | Enterectomy, resection of small intestine; with enterostomy | | | |
| 44130 | Enteroenterostomy, anastomosis of intestine, with or without cutaneous enterostomy (separate procedure) | | | |
| 44139 | Mobilization (take-down) of splenic flexure performed in conjunction with partial colectomy (List separately in addition to primary procedure) | | | |
| 44140 | Colectomy, partial; with anastomosis | | | |
| 44141 | Colectomy, partial; with skin level cecostomy or colostomy | | | |
| 44143 | Colectomy, partial; with end colostomy and closure of distal segment (Hartmann type procedure) | | | |
| 44144 | Colectomy, partial; with resection, with colostomy or ileostomy and creation of mucofistula | | | |
| 44145 | Colectomy, partial; with coloproctostomy (low pelvic anastomosis) | | | |
| 44146 | Colectomy, partial; with coloproctostomy (low pelvic anastomosis), with colostomy | | | |
| 44147 | Colectomy, partial; abdominal and transanal approach | | | |
| 44150 | Colectomy, total, abdominal, without proctectomy; with ileostomy or ileoproctostomy | | | |
| 44151 | Colectomy, total, abdominal, without proctectomy; with continent ileostomy | | | |
| 44155 | Colectomy, total, abdominal, with proctectomy; with ileostomy | | | |
| 44156 | Colectomy, total, abdominal, with proctectomy; with continent ileostomy | | | |
| 44157 | Colectomy, total, abdominal, with proctectomy; with ileoanal anastomosis, includes loop ileostomy, and rectal mucosectomy, when performed | | | |
| 44158 | Colectomy, total, abdominal, with proctectomy; with ileoanal anastomosis, creation of ileal reservoir (S or J), includes loop ileostomy, and rectal mucosectomy, when performed | | | |
| 44160 | Colectomy, partial, with removal of terminal ileum with ileocolostomy | | | |
| 44187 | Laparoscopy, surgical; ileostomy or jejunostomy, non-tube | | | |
| 44188 | Laparoscopy, surgical, colostomy or skin level cecostomy | | | |
| 44202 | Laparoscopy, surgical; enterectomy, resection of small intestine, single resection and anastomosis | | | |
| 44203 | Laparoscopy, surgical; each additional small intestine resection and anastomosis (List separately in addition to code for primary procedure) | | | |
| 44204 | Laparoscopy, surgical; colectomy, partial, with anastomosis | | | |
| 44205 | Laparoscopy, surgical; colectomy, partial, with removal of terminal ileum with ileocolostomy | | | |
| 44206 | Laparoscopy, surgical; colectomy, partial, with end colostomy and closure of distal segment (Hartmann type procedure) | | | |
| 44207 | Laparoscopy, surgical; colectomy, partial, with anastomosis, with coloproctostomy (low pelvic anastomosis) | | | |
| 44208 | Laparoscopy, surgical; colectomy, partial, with anastomosis, with coloproctostomy (low pelvic anastomosis) with colostomy | | | |
| 44210 | Laparoscopy, surgical; colectomy, total, abdominal, without proctectomy, with ileostomy or ileoproctostomy | | | |
| 44211 | Laparoscopy, surgical; colectomy, total, abdominal, with proctectomy, with ileoanal anastomosis, creation of ileal reservoir (S or J), with loop ileostomy, includes rectal mucosectomy, when performed | | | |
| 44212 | Laparoscopy, surgical; colectomy, total, abdominal, with proctectomy, with ileostomy | | | |
| 44213 | Laparoscopy, surgical, mobilization (take-down) of splenic flexure performed in conjunction with partial colectomy (List separately in addition to primary procedure) | | | |
| 44227 | Laparoscopy, surgical, closure of enterostomy, large or small intestine, with resection and anastomosis | | | |
| 44310 | Ileostomy or jejunostomy, non-tube | | | |
| 44312 | Revision of ileostomy; simple (release of superficial scar) (separate procedure) | | | |
| 44314 | Revision of ileostomy; complicated (reconstruction in-depth) (separate procedure) | | | |
| 44316 | Continent ileostomy (Kock procedure) (separate procedure) | | | |
| 44320 | Colostomy or skin level cecostomy; | | | |
| 44340 | Revision of colostomy; simple (release of superficial scar) (separate procedure) | | | |
| 44345 | Revision of colostomy; complicated (reconstruction in-depth) (separate procedure) | | | |
| 44346 | Revision of colostomy; with repair of paracolostomy hernia (separate procedure) | | | |
| 44615 | Intestinal stricturoplasty (enterotomy and enterorrhaphy) with or without dilation, for intestinal obstruction | | | |
| 44620 | Closure of enterostomy, large or small intestine; | | | |
| 44625 | Closure of enterostomy, large or small intestine; with resection and anastomosis other than colorectal | | | |
| 44626 | Closure of enterostomy, large or small intestine; with resection and colorectal anastomosis (eg, closure of Hartmann type procedure) | | | |
| 44640 | Closure of intestinal cutaneous fistula | | | |
| 44650 | Closure of enteroenteric or enterocolic fistula | | | |
| 44660 | Closure of enterovesical fistula; without intestinal or bladder resection | | | |
| 44661 | Closure of enterovesical fistula; with intestine and/or bladder resection | | | |
| 45000 | Transrectal drainage of pelvic abscess | | | |
| 45000 | Transrectal drainage of pelvic abscess | | | |
| 45005 | Incision and drainage of submucosal abscess, rectum | | | |
| 45020 | Incision and drainage of deep supralevator, pelvirectal, or retrorectal abscess | | | |
| 45020 | Incision and drainage of deep supralevator, pelvirectal, or retrorectal abscess | | | |
| 45110 | Proctectomy; complete, combined abdominoperineal, with colostomy | | | |
| 45111 | Proctectomy; partial resection of rectum, transabdominal approach | | | |
| 45112 | Proctectomy, combined abdominoperineal, pull-through procedure (eg, colo-anal anastomosis) | | | |
| 45113 | Proctectomy, partial, with rectal mucosectomy, ileoanal anastomosis, creation of ileal reservoir (S or J), with or without loop ileostomy | | | |
| 45114 | Proctectomy, partial, with anastomosis; abdominal and transsacral approach | | | |
| 45116 | Proctectomy, partial, with anastomosis; transsacral approach only (Kraske type) | | | |
| 45119 | Proctectomy, combined abdominoperineal pull-through procedure (eg, colo-anal anastomosis), with creation of colonic reservoir (eg, J-pouch), with diverting enterostomy when performed | | | |
| 45123 | Proctectomy, partial, without anastomosis, perineal approach | | | |
| 45136 | Excision of ileoanal reservoir with ileostomy | | | |
| 45150 | Division of stricture of rectum | | | |
| 45395 | Laparoscopy, surgical; proctectomy, complete, combined abdominoperineal, with colostomy | | | |
| 45397 | Laparoscopy, surgical; proctectomy, combined abdominoperineal pull-through procedure (eg, colo-anal anastomosis), with creation of colonic reservoir (eg, J-pouch), with diverting enterostomy, when performed | | | |
| 45399 | Unlisted laparoscopy procedure, colon | | | |
| 45499 | Unlisted laparoscopy procedure, rectum | | | |
| 45500 | Proctoplasty; for stenosis | | | |
| 45563 | Exploration, repair, and presacral drainage for rectal injury; with colostomy | | | |
| 45800 | Closure of rectovesical fistula; | | | |
| 45805 | Closure of rectovesical fistula; with colostomy | | | |
| 45820 | Closure of rectourethral fistula; | | | |
| 45825 | Closure of rectourethral fistula; with colostomy | | | |
| 45910 | Dilation of rectal stricture (separate procedure) under anesthesia other than local | | | |
| 45999 | Unlisted procedure, rectum | | | |
| 46020 | Placement of seton | | | |
| 46040 | Incision and drainage of ischiorectal and/or perirectal abscess (separate procedure) | | | |
| 46045 | Incision and drainage of intramural, intramuscular, or submucosal abscess, transanal, under anesthesia | | | |
| 46050 | Incision and drainage, perianal abscess, superficial | | | |
| 46060 | Incision and drainage of ischiorectal or intramural abscess, with fistulectomy or fistulotomy, submuscular, with or without placement of seton | | | |
| 46080 | Sphincterotomy, anal, division of sphincter (separate procedure) | | | |
| 46200 | Fissurectomy, including sphincterotomy, when performed | | | |
| 46270 | Surgical treatment of anal fistula (fistulectomy/fistulotomy); subcutaneous | | | |
| 46275 | Surgical treatment of anal fistula (fistulectomy/fistulotomy); intersphincteric | | | |
| 46280 | Surgical treatment of anal fistula (fistulectomy/fistulotomy); transsphincteric, suprasphincteric, extrasphincteric or multiple, including placement of seton, when performed | | | |
| 46285 | Surgical treatment of anal fistula (fistulectomy/fistulotomy); second stage | | | |
| 46288 | Closure of anal fistula with rectal advancement flap | | | |
| 46700 | Anoplasty, plastic operation for stricture; adult | | | |
| 46706 | Repair of anal fistula with fibrin glue | | | |
| 46707 | Repair of anorectal fistula with plug (eg, porcine small intestine submucosa [SIS]) | | | |
| 46710 | Repair of ileoanal pouch fistula/sinus (eg, perineal or vaginal), pouch advancement; transperineal approach | | | |
| 46712 | Repair of ileoanal pouch fistula/sinus (eg, perineal or vaginal), pouch advancement; combined transperineal and transabdominal approach | | | |
| 46999 | Unlisted procedure, anus | | | |
| 57307 | Closure of rectovaginal fistula | | | |
|  | | | | |
| **ICD-9-CM** | | | | |
| 17.3 | Laproscopic partial excision of large intestine | | | |
| 45.0 | Enterotomy | | | |
| 45.6 | Other excision of small intestine | | | |
| 45.61 | Multiple segmental resection of small intestine | | | |
| 45.62 | Other partial resection of small intestine | | | |
| 45.63 | Total removal of small intestine | | | |
| 45.7 | Open and partial excision of large intestine | | | |
| 45.71 | Open and other multiple segmental resection of large intestine | | | |
| 45.72 | Open and other cecectomy | | | |
| 45.73 | Open and other right hemicolectomy | | | |
| 45.74 | Open and other resection of transverse colon | | | |
| 45.75 | Open and other left hemicolectomy | | | |
| 45.76 | Open and other sigmoidectomy | | | |
| 45.79 | Other and unspecified partial excision of large intestine | | | |
| 45.8 | Total intra-abdominal colectomy | | | |
| 45.81 | Laparoscopic total intra-abdominal colectomy | | | |
| 45.82 | Open total intra-abdominal colectomy | | | |
| 45.83 | Other and unspecified total intra-abdominal colectomy | | | |
| 45.9 | Intestinal anastomosis | | | |
| 46.0 | Exteriorization of intestine | | | |
| 46.01 | Exteriorization of small intestine | | | |
| 46.02 | Resection of exteriorized segment of small intestine | | | |
| 46.03 | Exteriorization of large intestine | | | |
| 46.04 | Resection of exteriorized segment of large intestine | | | |
| 46.1 | Colostomy | | | |
| 46.2 | Ileostomy | | | |
| 46.31 | Delayed opening of other enterostomy | | | |
| 46.39 | Other enterostomy | | | |
| 46.72 | Closure of fistula of duodenum | | | |
| 46.74 | Closure of fistula of small intestine, except duodenum | | | |
| 46.76 | Closure of fistula of large intestine | | | |
| 48.0 | Proctotomy | | | |
| 48.1 | Proctostomy | | | |
| 48.4 | Pull-through resection of rectum | | | |
| 48.5 | Abdominoperineal resection of rectum | | | |
| 48.6 | Other resection of rectum | | | |
| 48.72 | Closure of proctostomy | | | |
| 48.73 | Closure of other rectal fistula | | | |
| 48.74 | Rectorectostomy | | | |
| 48.79 | Other repair of rectum | | | |
| 48.91 | Incision of rectal stricture | | | |
| 48.93 | Repair of perirectal fistula | | | |
| 48.99 | Other operations on rectum and perirectal tissue | | | |
| 49.1 | Incision or excision of anal fistula | | | |
| 49.11 | Anal fistulotomy | | | |
| 49.12 | Anal fistulectomy | | | |
| 49.5 | Division of anal sphincter | | | |
| 49.51 | Left lateral anal sphincterotomy | | | |
| 49.52 | Posterior anal sphincterotomy | | | |
| 49.59 | Other anal sphincterotomy | | | |
| 49.6 | Excision of anus | | | |
| 49.73 | Closure of anal fistula | | | |
| 49.93 | Other incision of anus | | | |
| 49.99 | Other operations on anus | | | |
| 70.73 | Repair of rectovaginal fistula | | | |
|  | | | | |
| **ICD-10-CM** | | | | |
| **Surgery location** | | **Surgery location description** | **Surgery type** | **Surgery type description** |
| 0Dx8xxx | | Small Intestine | 0D1xxxx | Bypass |
| 0Dx9xxx | | Duodenum | 0D5xxxx | Destruction |
| 0DxAxxx | | Jejunum | 0D7xxxx | Dilation |
| 0DxBxxx | | Ileum | 0D8Rxxx | Division of anal sphincter |
| 0DxCxxx | | Ileocecal Valve | 0D9xxxx | Drainage |
| 0DxExxx | | Large Intestine | 0DBxxxx | Excision |
| 0DxFxxx | | Large Intestine, Right | 0DCxxxx | Extirpation |
| 0DxGxxx | | Large Intestine, Left | 0DDxxxx | Extraction |
| 0DxHxxx | | Cecum | 0DFxxxx | Fragmentation |
| 0DxKxxx | | Ascending Colon | 0DHxxxx | Insertion |
| 0DxLxxx | | Transverse Colon | 0DLxxxx | Occlusion |
| 0DxMxxx | | Descending Colon | 0DMxxxx | Reattachment |
| 0DxNxxx | | Sigmoid Colon | 0DNxxxx | Release |
| 0DxPxxx | | Rectum | 0DPxxxx | Removal |
| 0DxQxxx | | Anus | 0DQxxxx | Repair |
| 0DxRxxx | | Anal Sphincter | 0DRRxxx | Replacement of anal sphincter |
|  | |  | 0DSxxxx | Reposition |
|  | |  | 0DTxxxx | Resection |
|  | |  | 0DUxxxx | Supplement |
|  | |  | 0DVxxxx | Restriction |
|  | |  | 0DWxxxx | Revision |
|  | |  | 0DXxxxx | Transfer |

**Abbreviations:** ICD-9-CM, International Classification of Diseases, 9^th^ edition, Clinical Modification; ICD-10-CM, International Classification of Diseases, 10^th^ edition, Clinical Modification

**Supplemental Table 3. Patient characteristics before and after matching**

|  | Before matching | | | After matching | | |
| --- | --- | --- | --- | --- | --- | --- |
|  | Adalimumab  N = 4,023 | Vedolizumab  N = 469 | SMD | Adalimumab  N = 461 | Vedolizumab  N = 461 | SMD |
| **Demographics as of the index date** | | | | | | |
| Age, mean ± SD (years) | 39.91 ± 14.53 | 43.45 ± 14.10 | 0.248 | 43.30 ± 15.01 | 43.34 ± 14.09 | 0.003 |
| Male, n (%) | 1,958 (48.7%) | 211 (45.0%) | 0.074 | 216 (46.9%) | 210 (45.6%) | 0.026 |
| US region of residence, n (%) |  |  | 0.132 |  |  | 0.166 |
| Northeast | 777 (19.3%) | 100 (21.3%) |  | 75 (16.3%) | 99 (21.5%) |  |
| North Central | 904 (22.5%) | 119 (25.4%) |  | 115 (24.9%) | 116 (25.2%) |  |
| South | 1,852 (46.0%) | 187 (39.9%) |  | 196 (42.5%) | 185 (40.1%) |  |
| West | 488 (12.1%) | 62 (13.2%) |  | 75 (16.3%) | 60 (13.0%) |  |
| **CCI, mean ± SD** | 0.34 ± 0.80 | 0.58 ± 1.24 | 0.229 | 0.50 ± 1.06 | 0.52 ± 1.09 | 0.018 |
| **Disease characteristics during the baseline period, n (%)** | | | | | | |
| Abdominal pain | 2,133 (53.0%) | 215 (45.8%) | 0.144 | 200 (43.4%) | 209 (45.3%) | 0.039 |
| Anemia | 964 (24.0%) | 120 (25.6%) | 0.038 | 104 (22.6%) | 115 (24.9%) | 0.056 |
| Anxiety | 493 (12.3%) | 83 (17.7%) | 0.153 | 71 (15.4%) | 80 (17.4%) | 0.053 |
| Diarrhea | 1,516 (37.7%) | 151 (32.2%) | 0.115 | 142 (30.8%) | 150 (32.5%) | 0.037 |
| Rectal bleeding | 737 (18.3%) | 56 (11.9%) | 0.179 | 55 (11.9%) | 56 (12.1%) | 0.007 |
| Respiratory or other chest symptoms | 716 (17.8%) | 104 (22.2%) | 0.110 | 106 (23.0%) | 100 (21.7%) | 0.031 |
| Gastrointestinal hemorrhage | 481 (12.0%) | 31 (6.6%) | 0.185 | 35 (7.6%) | 31 (6.7%) | 0.034 |
| Symptoms of the abdomen and pelvis | 2,427 (60.3%) | 253 (53.9%) | 0.129 | 241 (52.3%) | 247 (53.6%) | 0.026 |
| CD-related complications (any) | 922 (22.9%) | 100 (21.3%) | 0.038 | 101 (21.9%) | 97 (21.0%) | 0.021 |
| Stricture (intestinal) | 560 (13.9%) | 66 (14.1%) | 0.004 | 63 (13.7%) | 63 (13.7%) | 0.000 |
| Perianal fistula | 211 (5.2%) | 25 (5.3%) | 0.004 | 22 (4.8%) | 25 (5.4%) | 0.030 |
| Perianal abscess | 161 (4.0%) | 13 (2.8%) | 0.068 | 20 (4.3%) | 13 (2.8%) | 0.082 |
| Internal fistula | 151 (3.8%) | 15 (3.2%) | 0.030 | 15 (3.3%) | 15 (3.3%) | 0.000 |
| Internal abscess | 66 (1.6%) | 3 (0.6%) | 0.094 | 3 (0.7%) | 3 (0.7%) | 0.000 |
| CD location as of the index date |  |  |  |  |  |  |
| Small intestine | 991 (24.6%) | 94 (20.0%) | 0.110 | 131 (28.4%) | 93 (20.2%) | 0.193 |
| Large intestine | 736 (18.3%) | 90 (19.2%) | 0.023 | 80 (17.4%) | 89 (19.3%) | 0.050 |
| Small and large intestine | 744 (18.5%) | 89 (19.0%) | 0.012 | 87 (18.9%) | 88 (19.1%) | 0.006 |
| Unspecified | 1,676 (41.7%) | 204 (43.5%) | 0.037 | 177 (38.4%) | 199 (43.2%) | 0.097 |
| **Treatment history during the baseline period, n (%)** | | | | | | |
| Systemic corticosteroids | 2,600 (64.6%) | 267 (56.9%) | 0.158 | 243 (52.7%) | 263 (57.0%) | 0.087 |
| Opioids | 1,592 (39.6%) | 192 (40.9%) | 0.028 | 181 (39.3%) | 184 (39.9%) | 0.013 |
| Aminosalicylates | 1,522 (37.8%) | 114 (24.3%) | 0.295 | 105 (22.8%) | 113 (24.5%) | 0.041 |
| Immunosuppressants | 1,136 (28.2%) | 103 (22.0%) | 0.145 | 92 (20.0%) | 102 (22.1%) | 0.053 |
| **HRU during the baseline period** | | | | | | |
| All-cause HRU: Proportion of patients with any, n (%) |  |  |  |  |  |  |
| Inpatient admissions | 917 (22.8%) | 114 (24.3%) | 0.036 | 99 (21.5%) | 109 (23.6%) | 0.052 |
| ER visits | 1,220 (30.3%) | 154 (32.8%) | 0.054 | 130 (28.2%) | 148 (32.1%) | 0.085 |
| CD-related HRU: Proportion of patients with any, n (%) |  |  |  |  |  |  |
| Inpatient admissions | 819 (20.4%) | 105 (22.4%) | 0.050 | 90 (19.5%) | 100 (21.7%) | 0.054 |
| ER visits | 483 (12.0%) | 71 (15.1%) | 0.092 | 49 (10.6%) | 66 (14.3%) | 0.112 |
| **All-cause treatment costs (2020 US dollars)** | | | | | | |
| Mean ± SD | 2,815 ± 4,120 | 4,024 ± 9,295 | 0.168 | $2,801 ± $6,187 | $3,661 ± $7,139 | 0.129 |
| Median | 1,443 | 1,355 |  | 926 | 1,314 |  |
| IQR | (241, 4,048) | (112, 4,169) |  | (132, 3,213) | (105, 4,097) |  |

**Abbreviations:** CCI, Charlson Comorbidity Index; CD, Crohn’s disease; ER, emergency room; HRU, health resource utilization; IQR, interquartile range; SD, standard deviation; SMD, standardized mean difference; US, United States.

**Supplemental Table 4. Healthcare resource use in the follow-up period**

|  | **Adalimumab N = 461** | **Vedolizumab N = 461** | **P-value** |
| --- | --- | --- | --- |
| **All-cause HRU: Number of admissions or visits, mean ± SD** |  |  |  |
| Inpatient admissions | 0.28 ± 0.91 | 0.40 ± 0.92 | 0.008 * |
| ER visits | 0.65 ± 1.53 | 1.09 ± 2.07 | < 0.001 * |
| **CD-related HRU: Number of admissions or visits, mean ± SD** |  |  |  |
| Inpatient admissions | 0.26 ± 0.83 | 0.35 ± 0.88 | 0.038 * |
| ER visits | 0.24 ± 0.81 | 0.47 ± 1.41 | 0.005 * |

**Abbreviations:** CD, Crohn’s disease; ER, emergency room; HRU, health resource utilization; SD, standard deviation.

***** denotes statistical significance (p<0.05)

**Supplemental Table 5. Healthcare costs in the follow-up period**

|  | **Adalimumab N = 461** | **Vedolizumab N = 461** | **P-value** |
| --- | --- | --- | --- |
| **All-cause healthcare costs (2020 USD)** |  |  |  |
| Total costs |  |  | 0.466 |
| Mean ± SD | 90,113 ± 95,382 | 95,955 ± 66,022 |  |
| Median | 76,802 | 75,067 |  |
| IQR | (61,066, 96,892) | (56,060, 111,575) |  |
| Medical costs |  |  | < 0.001 * |
| Mean ± SD | 27,240 ± 94,306 | 32,441 ± 50,212 |  |
| Median | 7,554 | 15,328 |  |
| IQR | (2,691, 19,926) | (6,408, 37,715) |  |
| Treatment costs |  |  | 0.008 * |
| Mean ± SD | 62,873 ± 32,146 | 63,514 ± 41,371 |  |
| Median | 62,506 | 52,794 |  |
| IQR | (45,401, 75,544) | (43,344, 75,486) |  |
| **CD-related healthcare costs (2020 USD)** |  |  |  |
| Total costs |  |  | 0.349 |
| Mean ± SD | 73,707 ± 52,246 | 77,095 ± 53,784 |  |
| Median | 67,427 | 59,938 |  |
| IQR | (50,867, 82,673) | (49,699, 91,731) |  |
| Medical costs |  |  | < 0.001 * |
| Mean ± SD | 15,284 ± 47,676 | 19,437 ± 34,135 |  |
| Median | 2,775 | 6,430 |  |
| IQR | (703, 10,477) | (3,002, 19,611) |  |
| Treatment costs |  |  | 0.001 * |
| Mean ± SD | 58,424 ± 30,712 | 57,658 ± 39,511 |  |
| Median | 58,191 | 50,014 |  |
| IQR | (40,566, 71,979) | (38,325, 66,971) |  |

**Abbreviation:** CD, Crohn’s disease; IQR, interquartile range; SD, standard deviation; USD, United States dollars.

***** denotes statistical significance (p<0.05)
